# Supplementary material for: Analysing multimodal data that have been collected using photovoice as a research method
Source: BMJ Open. 2023 Apr 17;13(4):e068289. doi: 10.1136/bmjopen-2022-068289 (PMC10111907; doi:10.1136/bmjopen-2022-068289)
Supplement: Author's manuscript [file bmjopen-2022-068289.draft_revisions.pdf]

BMJ Open

BMJ Open is committed to open peer review. As part of this commitment we make the peer review history of every article we publish publicly available.

When an article is published we post the peer reviewers' comments and the authors' responses online. We also post the versions of the paper that were used during peer review. These are the versions that the peer review comments apply to.

The versions of the paper that follow are the versions that were submitted during the peer review process. They are not the versions of record or the final published versions. They should not be cited or distributed as the published version of this manuscript.

BMJ Open is an open access journal and the full, final, typeset and author-corrected version of record of the manuscript is available on our site with no access controls, subscription charges or pay-per-view fees (http://bmjopen.bmj.com).

If you have any questions on BMJ Open's open peer review process please email info.bmjopen@bmj.com

BMJ Open

Analysing multi modal data that has been collected using photovoice as a research method

Journal:	*BMJ Open*
Manuscript ID	bmjopen-2022-068289.R1
Article Type:	Communication
Date Submitted by the Author:	13-Oct-2022
Complete List of Authors:	Mooney, Roisin; University of Oxford, Psychiatry<br>Bhui , Kamaldeep; University of Oxford,<br>Project Team , Co-Pact ; Oxford University
**Primary Subject Heading**:	Health services research
Secondary Subject Heading:	Qualitative research, Mental health, Communication
Keywords:	QUALITATIVE RESEARCH, MENTAL HEALTH, PSYCHIATRY

SCHOLARONE™
Manuscripts

I, the Submitting Author has the right to grant and does grant on behalf of all authors of the Work (as defined in the below author licence), an exclusive licence and/or a non-exclusive licence for contributions from authors who are: i) UK Crown employees; ii) where BMJ has agreed a CC-BY licence shall apply, and/or iii) in accordance with the terms applicable for US Federal Government officers or employees acting as part of their official duties; on a worldwide, perpetual, irrevocable, royalty-free basis to BMJ Publishing Group Ltd ("BMJ") its licensees and where the relevant Journal is co-owned by BMJ to the co-owners of the Journal, to publish the Work in BMJ Open and any other BMJ products and to exploit all rights, as set out in our licence.

The Submitting Author accepts and understands that any supply made under these terms is made by BMJ to the Submitting Author unless you are acting as an employee on behalf of your employer or a postgraduate student of an affiliated institution which is paying any applicable article publishing charge ("APC") for Open Access articles. Where the Submitting Author wishes to make the Work available on an Open Access basis (and intends to pay the relevant APC), the terms of reuse of such Open Access shall be governed by a Creative Commons licence – details of these licences and which Creative Commons licence will apply to this Work are set out in our licence referred to above.

Other than as permitted in any relevant BMJ Author's Self Archiving Policies, I confirm this Work has not been accepted for publication elsewhere, is not being considered for publication elsewhere and does not duplicate material already published. I confirm all authors consent to publication of this Work and authorise the granting of this licence.

For peer review only

Analysing multi modal data that has been collected using photovoice as a research method

Dr Roisin Mooney & Prof. Kam Bhui, Co-Pact Project Team.

CHiMES Collaborative, Dept of Psychiatry, University of Oxford

Co-Pact Team; Doreen Joseph (Doreen.Joseph@psych.ox.ac.uk), Prof. Nusrat Husain (nusrat.husain@manchester.ac.uk), Prof. Rose McCabe (rose.mccabe@city.ac.uk), Prof. Paul McCrone (P.McCrone@greenwich.ac.uk), Dr Karen Newbigging (karen.newbigging@psych.ox.ac.uk), Prof. Raghu Ragahavan (rraghavan@dmu.ac.uk), Dr Clair Dempsey (clair.dempsey@psych.ox.ac.uk), Michelle Yeung (michelle.yeung@psych.ox.ac.uk)

Corresponding author: Dr Roisin Mooney

Roisin.mooney@psych.ox.ac.uk

CHiMES Collaborative

Deptartment of Psychiatry

University of Oxford

Warneford Hospital

Headington

Oxford

OX3 7JX

Abstract (121 words)

Creative arts practice can enhance the depth and quality of mental health research by capturing and foregrounding participants' lived experience. Creative methods are emotionally activating and promote multiple perspectives, tolerating ambiguities and uncertainties, which are shared and even celebrated. Methods such as photovoice use imagery to elucidate narratives that are not easily captured by more traditional interview based research techniques. However, the use of creative methods and participatory research remains novel as there is little guidance of how to navigate conceptual, practical, and analytical challenges. This paper considers these challenges, and puts forward practical and theory

For peer review only

informed recommendations, using as study of photovoice methods for investigating ethnic inequalities in the use of the mental health act (Co-Pact) as a case study.

Photovoice as a research method (246 words)

The use of photography and visual images as a form of research (photovoice) is an increasingly popular way to gather patient experiences that are overlooked, hidden or difficult to articulate. The purpose is to give 'voice' to experiences of injustice and adversity in a psychologically safe way. This knowledge can be used to inform the design of more inclusive policies, public services, and practice [1].

Grounded in feminist theory and empowerment, photovoice was first developed as a method to engage communities in sharing their lived experiences in the early 1990s, and was used in areas of health education, community activism, and public services [2]. In the last 30 years photovoice has been adapted for many different contexts [1][3]. Thus, the approach is gaining traction as a qualitative research method that enables non-verbal, emotional, pre-conscious, and creatives processes to be activated, in turn enabling difficult experiences to be surfaced and explored. Participation in photovoice has been demonstrated to empower participants [4], improve reflexivity [5], and promote a sense of personal accountability [6]. Creative methods generally, and photovoice specifically, can help to reduce power imbalances between researchers and research participants, by both parties interpreting the image and placing the participant's voice centre stage. This is especially important where there is a lack of trust, concerns about institutional harms, or lack of psychological safety (as is the case for ethnic minorities in contact with mental health services), and where stigma may also prevent openness.

Implementing photovoice (424 words)

Photovoice methods involve posing a question and asking people to represent their views or sentiments through photographs or photo-assignments, followed by a process of interpretation or photo-discussion. This can be done individually or in groups, eliciting both visual (photographs) and narrative data (participants' voices). There is no convention as to the number of photo-assignments or photo-discussions, nor the duration of meetings and ways of assembling, analysing and presenting the resulting data. The data collection and analysis may consist of a linear approach by which participants, take photographs, interpret them and share them with each other, and/or with researchers. The assumption is that material

For peer review only

is produced and unambiguously interpreted by the participants themselves, then presented for wider discussion to generate consensus. Alternatively, some researchers have adopted a more iterative and multi-layered approach whereby a series of photo-assignments, and related discussions are held, with evolving meanings and re-appraisal of motivations, affects, attitudes, and emergent interpretations over time [7]. This permits a multitude of perspectives which may be diverse and contradictory even, but represent valuable insights into lived experience and perspectives of importance to participants.

The photographs shared as part of the photovoice process provide a rich detailed insight as to how the participants experience, interact with and understand the world around them, enabling those viewing the images to see the world from the participants' perspective. Data captured in photographs goes beyond the visual description of the content of the photograph; for example, metaphors are often used to convey feelings or experiences that are difficult to put into words. Photographs may be interpreted subjectively and are likely therefore to give expression to aspects of experience that might not be recognised, acknowledged, or even shared in a group. There may be social norms to which individuals feel obliged to conform.

There are multiple levels of content, and hence, the analysis and interpretation must be appropriately nuanced. For example, the way a person chooses to compose the images, the subject matter, but also the way they engage with the task, the format (virtual or face to face) the timing, and types of camera used. All of these unspoken perspectives may emerge in and impact the interpretation and analysis. Furthermore, the form of picture development, black and white, sepia, or grained, and use of digital tools to modify photographs are all possible now. Undertaking the exercise in groups with other service users or peer researchers can also provide a more supportive and facilitative environment where critical reflection of the experiences and the potential for new understandings emerge from the discussion.

Embedding rigor to maintain research integrity (308 words)

As the photovoice approach gains popularity in part attributable to the significant flexibility it offers, it is important to ensure that the underpinning theory is understood and considered in both collecting data and the consequent analytic approach adopted [8]. The potential for experiential data to inform policy is progressive, and likely to be an important way of ensuring inclusive research that fully represents marginalised perspectives. However, the stages of moving through recruitment of participants, data collection, data storage, coding the

For peer review only

data, extracting information, analysis and dissemination are less well defined. This lack of guidance is helpful if the photograph is seen as an art product, subject to multiple interpretations and meaning-making as an essential attribute that engages and promotes eudemonic satisfaction. Indeed, art in part seeks to resist reductive classifications and analysis and open the viewer and group of viewers to interact around shared and unshared experiences and interpretation.

As creative processes are not reliant on language, nor time-pressured interviews, and more supportive of staged elicitation of views, experiences that are fragile and difficult to verbalise, overlooked, or hidden most of the time can emerge and be deepened during the research. Visual and experience-near narratives are continually available to be used in a variety of contexts, such as being curated for public display, or synthesised with evidence from other research methods that, if used alone, risk losing insights from marginalised groups. It is important to ensure clarity of process and document decisions, so that the contributions of participants are fully represented and are not lost, diluted or redefined in the analytic process and summary statements.

The interpretation process can involve other people which can lead to the generation of additional perspectives and layers of meaning. Understanding these different layers is critically important in the analysis phase, but also to ensure authenticity in attributions of similar or different meanings.

Analytic challenges arising from photovoice (1185 words)

Wang and Burris wrote "photographs are easy to gather but difficult to analyse and summarise because they yield an abundance of complex data that can be difficult to digest" (p375) [9]. Furthermore, it isn't only the photographs that have been taken that are presented and discussed by participants in photo-discussions, but also the photographs that they were unable to take, or chose not to take [10]. This notion of complexity is compounded by the lack of standardisation regarding how photovoice is implemented. The number of photographs being considered, the number of interactions between participants and researchers, and the number of interactions between different participants can vary greatly, all of which have an impact on the volume and quality of the data collected. Yet it is unknown what the optimal volume is, and what could indicate high quality photovoice data. These indicators may also vary according to the purpose and structure that photovoice is being

For peer review only

conducted within. For example, shorter captions to provide context to images, may be appropriate if there are multiple iterative group discussions. Whereas longer captions may be preferable if there are fewer interactions or discussions between the participants themselves or with the researcher.

Many traditions in qualitative psychological research have been generated based on oral or written data. Whilst the interpretation of speech or text can vary according to its intention, and the experiences of the person interpreting it, most words tend to have a clear and universally acknowledged meaning. Conversely interpreting photographs is subjective. Photographs may have multiple meanings and many different interpretations. Generally photographs have a public meaning (universally recognisable elements) and a private meaning (partial objects in the pictures that have an emotional origin and are difficult to express in words) [11][12]. It is important to understand how participants assign meaning to their images and create a space that enables them to communicate their private meaning to both the researchers and other participants. This process also permits the participant, the owner of the photograph, to retain control about what is communicated and what is silenced. The photos act as an elicitation tool, to help reflection, recall, and verbalisation of unexpressed views. Fostering collaboration between researchers and participants may minimise power imbalances, and elicit new, deeper understanding as opposed to the potentially rehearsed narratives that often manifest in traditional forms of data collection such as interviews and questionnaires.

The analysis of photovoice data typically includes inductive forms of analysis such as interpretative phenomenological analysis (IPA)[13][14][15], grounded theory [16][17][18] and thematic analysis [19][16][20]. Inductive approaches use the data as a starting point for the analysis as opposed to applying a deductive framework based on existing knowledge, however “we cannot conduct research without making assumptions” (p9) [21]. There are assumptions about how knowledge is created and communicated that underpin qualitative forms of analysis. It is important to understand how these assumptions manifest and the context within which the data was collected when deciding which form of analysis is appropriate. A core component of photovoice is the critical consciousness generated by the discussion of photographs [22], yet many of the psychological underpinnings in qualitative research are developed from a preoccupation with how an individual constructs and understands the world around them.

For peer review only

IPA aims to provide an in depth understanding of not only what an experience was like for participants but also how they made sense of it. Therefore, traditionally IPA involves two stages of interpretation; how the participants interpret their experiences, and how researchers interpret participants interpreting their experience [23]. The second stage risks diluting the voice of the participant. When adapting this approach for photovoice data some researchers add a data verification step [14]; others have analysed individual interviews about the photographs participants have taken using IPA, then looked for themes across these interviews [24] [13], or analysed transcripts of group discussions using IPA [15]. Whilst IPA may appear well suited for photovoice data owing to the ability view the world through the lens of the participant when examining photographs with the meaning ascribed to them by the participants themselves [25], this approach may not be well suited to large volumes of data that contains multiple layers (such as the captions for the individual photographs and the group discussions).

Grounded theory method is an iterative form of analysis that seeks to develop new explanations concerning basic social processes [26]. This form of analysis may be useful when there are multiple photovoice workshops or photo-assignment sessions, to enable the iterative development of a new theory grounded in lived experience [27]. The assumptions underpinning grounded theory also speak to participatory action research (PAR) methodology that collaborates with (rather than studies) people to develop new ways of seeing the world [28]. In practice this method may entail the development of a code book that can be applied to both visual and narrative data, alongside constant comparison and data verification with participants to ensure authenticity of the findings [17]. Interviews or focus groups may be conducted to elicit the meaning associated with the photographs, and the resulting transcripts analysed using grounded theory method [18]. This approach is particularly useful where there is capacity to hold a series of photovoice workshops or photo assignments and allow an iterative process.

Thematic analysis is described by Braun and Clarke as a method for identifying, analysing and reporting patterns within data [29]. The core aim of identifying patterns also renders this method suitable for both visual and narrative data. Thematic analysis has gained popularity over the last 15 years owing to its flexibility and accessibility for interdisciplinary researchers. The seven steps, transcription, familiarisation, coding, searching for themes,

For peer review only

reviewing themes, defining themes, and finalising themes; provide a rigorous framework that can be applied to large volumes of data in different contexts. Thematic analysis is commonly used within the psychological research approach where photovoice interviews have been employed with individuals [20][30][19]. When combining PAR and psychological research, the thematic approach is well suited to facilitating participants to collaborate in the analysis, by assisting in the identification of patterns across their data. Poly textual thematic analysis, is a further development of thematic analysis principles to incorporate visual data [31]. This approach allows researchers to analyse how photographs are interpreted by participants, as opposed to being analysed and interpreted by researchers. This approach appears optimal to ensure that photographs and narratives are never analysed independently of each other [7].

A qualitative scoping review of how photovoice was used in mental illness research found 9 studies, of which 7 analysed primary data [32]. The PAR approach and collaboration with the communities was central to primary studies. Grounded theory was the most cited analytical framework, however processes relating to the thematic analysis were often described in the absence of an explicitly stated analytical framework. Most studies involved participants in the analysis stage, but the majority were (n=7) were still perceived to be researcher-centric [32]. Within psychological research it has been proposed that the researchers and participants should analyse or interpret photographs independently, then compare their findings to generate holistic explanations and theorise phenomenon [33]. Of the 9 studies only 2 included formal analysis of the photographs.

Co-Pact: A Case Study (784 words)

Co-PACT is an NIHR funded study that seeks to combine photovoice and experience based co design [34] with the aim of reducing the ethnic inequalities experienced by those detained under the Mental Health Act (MHA). The use of photovoice to elucidate new perspectives and amplify the voices of marginalised populations in a creative and accessible manner renders it an optimal methodology to inform experience based co design meetings [35]. The intention of this study is to have an impact at a national policy level, by informing the current reform of the MHA. Photovoice data is being collected across eight NHS trusts from service

For peer review only

users who have experience of being detained under the MHA in the last 48 months. Recruitment for these workshops has been purposive to ensure that those from marginalised backgrounds are facilitated to engage with the research process and to ensure that a range of difference section types, demographics and experiences are captured.

We have linked photovoice to experience based co-design to ensure service users themselves (working alongside other stakeholders) will have the power or ability to change an intrinsically complex system, that has resulted in their unequal treatment. We want to ensure that we allow for their authentic and undiluted voice to be heard in spaces where those who have the power to change the system can not only hear it but also engage with it. When discussing the photographs that they provide participants critically reflect on their own situation and produce materials that can be used to plan, inform and motivate change [36]. It is important for our analysis to capture the critical consciousness that results from the group dynamics (not solely analysis of the visual data and associated captions), to inform experience based co-design workshops which will aim to elicit changes in local systems, and consequently inform national policy.

Our approach to photovoice consists of a series of three workshops (figure. 1), facilitated by researchers, delivered face to face or online according to participant preference.

[insert figure 1]

We have had many interdisciplinary workshops and discussions (inclusive of people who have experienced detention under the MHA) to refine our analytic approach to the photovoice data, whilst being cognisant of the tensions and challenges with the complexity, volume and depth of the data we have collected. In recognition that one of the aims of the study is to foreground lived experience, it is important to acknowledge that the analysis of the photographs begins with the participant's own interpretation of their images, developing captions and assigning meaning to their image. A second stage of analysis has also occurred with participants discussing their images in the third workshop and beginning to consider

For peer review only

similarities and differences in their experiences, identifying patterns across the data. Based on approaches taken by other researchers working with visual data, and the principles of poly textual thematic analysis [31] we propose the following steps (table 1);

Table 1: Stages of analysis for multi-modal data

Step 1- Understanding the sample	Extract demographic information from 'about me' diagrams and roadmaps into an excel sheet (Sheet 1) Initial extraction includes; Age, Gender, Ethnicity, Number of times sectioned, Most recent section type and Diagnosis. Fields may be added as patterns in the data are recognised, for example; Childhood trauma, age of onset and physical co-morbidities.
Step 2 – Organise the data	Organise data into a second excel spreadsheet (Sheet 2) containing the following headings; Image ID, Participant's caption, Image, Alternative Text Description (this will be generated by the research team, and will be a very basic narrative description, to assist the organisation of data), Mentions in transcript from the third workshop discussion, Similar Images and Participant Demographics. This will enable a framework analysis of the data and a way to organise the data according to different variables. Researchers should keep a log book and note any observations they make during the process, such as how a group of images look together, and feelings or messages that are conveyed or noticed.
Step 3 – Individual Case Analysis	Consider the images and captions for each participant, consider similarities and themes within the images and the overall narrative that they are sharing. Code both the visual content of the images and the captions relating to the image, as well as noting any observations about the group of images together.
Step 4 – Exploring interactions	Go through the transcripts from the group discussions where participants discuss their images as a group, these should read as photo essays with the photographs added to the transcripts in the relevant places. Extract any

For peer review only

	references to the images and add these to sheet 2, this should include any elements where multiple participants are discussing the same image.
Step 5 – Combining the individual and group analyses	Incorporate information from the transcripts to the existing case based analysis (Sheet 3). For a multi – site study, steps 2-5 should be done at a site level to allow an understanding of how the local context may impact the shared experiences.
Step 6- Theme generation	Review Sheet 2 (the data) and Sheet 3 (the codes) for all sites, alongside people with lived experience, to consider and develop how codes may be grouped together to develop higher order themes. It is important that within this process, if there is a voice or experience that doesn't fit the dominant narrative that this isn't disregarded or diluted in the process. We should seek to understand the exceptions, and the potential reasons for these differences as well as the main themes.
Step 7 – Theme iteration	Review higher order themes, and the image/caption combinations within those, alongside field notes from the workshops as well as group notes generated from the discussion. This is to ensure that there aren't elements of the group discussion that were important to the participants who were present in the room that are missed.
Step 8 – Dissemination	Ensure that the outputs are used to motivate change, this should entail activities beyond publishing lay summaries and academic journal articles, such as presenting themes at co-design meetings and hosting public exhibitions of the images in collaboration with the participants.

For peer review only

When analysing photovoice data it is also important to remember the potential therapeutic impact on the participants from taking part in these workshops, and how this impacts the information they are sharing. The creative process, may have an impact on how the participants themselves relate to the experiences they are sharing, offering new insights as opposed to a rehearsed narrative. For marginalised communities, photovoice may be a more accessible way to part take in research, thus it is possible that the participants are very research naieve, not used to sharing their experiences and find benefit in participation.

We propose that the following questions are considered prior to analysis in future photovoice studies;

- What does the data consist of?
    - How many interactions are there between the participant and the research team?
    - How many images are there per participant?
    - How detailed is the accompanying narrative?
- What is the relationship between the participants and those who facilitated the workshops?
- Is there a group narrative, individual narrative or combination of both?
- Who is analysing the data?
- What is the analysis informing? For what purpose is the study being conducted?

Methodological advances in this area will only be possible if we continue to innovate, document and feedback our experiences of analysing visual and narrative data in combination. The development of creative, participatory methodologies such as photovoice are needed to further enable marginalised populations to contribute meaningfully towards research, and inform future innovations in health care.

Conclusion (98 words)

Photovoice is a creative way of enabling participants to share difficult experiences. Initially developed for the purpose of engaging communities and motivating social justice, there are a wide range of purposes for which photovoice has now been adopted. Researchers have used photovoice to elicit the narratives of marginalised populations whose voices are often silenced. The flexibility in how photovoice is implemented renders numerous possibilities and challenges when analysing the resulting data. For researchers it is important to be transparent about the steps taken when processing the data that participants have generated and to share how decisions were informed.

For peer review only

Authors contributions:

RM drafted the initial manuscript that was revised by KB, and then continual revisions made collaboratively.

The content of this article reflects conversations held by the Co-Pact team, a project for which KB and RM are Co-PIs.

Funding Statement

This study/project is funded by the National Institute for Health Research (NIHR) Policy Research Programme (NIHR201704). The views expressed are those of the authors and not necessarily those of the NIHR or the Department of Health and Social Care.

Competing interests' statement

Nothing to declare

Figure caption

Page 8 - Figure 1: The photovoice workshop process adopted in Co-pact

For peer review only

1 Halvorsrud K, Rhodes J, Webster GM, *et al.* Photovoice as a promising public engagement approach: capturing and communicating ethnic minority people's lived experiences of severe mental illness and its treatment. *BMJ Open Qual* 2019;**8**:e000665. doi:10.1136/bmjoq-2019-000665

2 Wang C, Burris MA. Empowerment through Photo Novella: Portraits of Participation. *Health Educ Q* 1994;**21**:171–86. doi:10.1177/109019819402100204

3 Halvorsrud K, Eylem O, Mooney R, *et al.* Identifying evidence of the effectiveness of photovoice: a systematic review and meta-analysis of the international healthcare literature. *J Public Health* Published Online First: 5 April 2021.https://doi.org/10.1093/pubmed/fdab074 (accessed 6 Sep 2021).

4 Budig K, Diez J, Conde P, *et al.* Photovoice and empowerment: evaluating the transformative potential of a participatory action research project. *BMC Public Health* 2018;**18**:432. doi:10.1186/s12889-018-5335-7

5 Hodgetts D, Chamberlain K, Groot S. 20 Reflections on the visual in community research and action. *Vis Methods Psychol Using Interpret Images Qual Res* 2012;:299.

6 Carlson ED, Engebretson J, Chamberlain RM. Photovoice as a Social Process of Critical Consciousness. *Qual Health Res* 2006;**16**:836–52. doi:10.1177/1049732306287525

7 Törnbom K, Lundälv J, Palstam A, *et al.* "My life after stroke through a camera lens"- A photovoice study on participation in Sweden. *PLOS ONE* 2019;**14**:e0222099. doi:10.1371/journal.pone.0222099

8 Liebenberg L. Thinking Critically About Photovoice: Achieving Empowerment and Social Change. *Int J Qual Methods* 2018;**17**:1609406918757631. doi:10.1177/1609406918757631

9 Wang C, Burris MA. Photovoice: concept, methodology, and use for participatory needs assessment. *Health Educ Behav Off Publ Soc Public Health Educ* 1997;**24**:369–87. doi:10.1177/109019819702400309

10 Hodgetts D, Chamberlain K, Radley A. Considering Photographs Never Taken During Photo-production Projects. *Qual Res Psychol* 2007;**4**:263–80. doi:10.1080/14780880701583181

11 Rania N, Migliorini L, Rebora S, *et al.* Photovoice and Interpretation of Pictures in a Group Discussion: A Community Psychology Approach. *Qual Res Psychol* 2015;**12**:382–96. doi:10.1080/14780887.2015.1019597

12 Warren S. Photography and voice in critical qualitative management research. *Account Audit Account J* 2005;**18**:861–82. doi:10.1108/09513570510627748

13 annadadswell A. Interpretative phenomenological analysis and photovoice methods in participatory therapeutic contexts. ARU Particip. Res. 2021.https://aruparticipatoryresearch.wordpress.com/2021/03/25/interpretative-phenomenological-analysis-and-photovoice-methods-in-participatory-therapeutic-contexts/ (accessed 25 Feb 2022).

For peer review only

14 Tanhan A, Strack RW. Online photovoice to explore and advocate for Muslim biopsychosocial spiritual wellbeing and issues: Ecological systems theory and ally development. *Curr Psychol* 2020;**39**:2010–25. doi:10.1007/s12144-020-00692-6

15 Cosgrove D, Bozlak C, Reid P. Service Barriers for Gender Nonbinary Young Adults: Using Photovoice to Understand Support and Stigma. *Affilia* 2021;**36**:220–39. doi:10.1177/0886109920944535

16 Latz AO, Mulvihill TM. *Photovoice Research in Education and Beyond: A Practical Guide from Theory to Exhibition*. New York: : Routledge 2017. doi:10.4324/9781315724089

17 Cabassa LJ, Nicasio A, Whitley R. Picturing recovery: a photovoice exploration of recovery dimensions among people with serious mental illness. *Psychiatr Serv Wash DC* 2013;**64**:837–42. doi:10.1176/appi.ps.201200503

18 Santos MAF, Lopes MAP, Botelho MAR. Photovoice as a Method of Data Collection in the Study of Motherhood Over the Age of 35: The Power of Images. In: Costa AP, Reis LP, Souza FN de, *et al.*, eds. *Computer Supported Qualitative Research*. Cham: : Springer International Publishing 2018. 316–26. doi:10.1007/978-3-319-61121-1_27

19 Glaw X, Inder K, Kable A, *et al.* Visual Methodologies in Qualitative Research: Autophotography and Photo Elicitation Applied to Mental Health Research. *Int J Qual Methods* 2017;**16**:1609406917748215. doi:10.1177/1609406917748215

20 Edwards LB, Greeff LE. A descriptive qualitative study of childhood cancer challenges in South Africa: Thematic analysis of 68 photovoice contributions. *South Afr J Oncol* 2017;**1**:8. doi:10.4102/sajo.v1i0.14

21 Rohleder P, Lyons AC. *Qualitative Research in Clinical and Health Psychology*. Macmillan International Higher Education 2014.

22 Freire P. Pedagogy of the Oppressed. 1968. *Trans Myra Bergman Ramos N Y Herder* 1972.

23 Smith JA. Evaluating the contribution of interpretative phenomenological analysis. *Health Psychol Rev* 2011;**5**:9–27. doi:10.1080/17437199.2010.510659

24 Keating F. Black men’s conversations about mental health through photos. *Qual Soc Work* 2021;**20**:755–72. doi:10.1177/1473325020922293

25 Brunsden V, Goatcher J. Reconfiguring Photovoice for Psychological Research. *Ir J Psychol* 2007;**28**:43–52. doi:10.1080/03033910.2007.10446247

26 Charmaz K. Reconstructing grounded theory. *SAGE Handb Soc Res Methods* 2008;:461–78.

27 Freedman DA, Pitner RO, Powers MCF, *et al.* Using Photovoice to Develop a Grounded Theory of Socio-Environmental Attributes Influencing the Health of Community Environments. *Br J Soc Work* 2014;**44**:1301–21. doi:10.1093/bjsw/bcs173

For peer review only

28 Bradbury H, Reason P. Action Research: An Opportunity for Revitalizing Research Purpose and Practices. *Qual Soc Work* 2003;**2**:155–75. doi:10.1177/1473325003002002003

29 Using thematic analysis in psychology: Qualitative Research in Psychology: Vol 3, No 2. https://www.tandfonline.com/doi/abs/10.1191/1478088706QP063OA (accessed 25 Feb 2022).

30 Chew HSJ, Lopez V. Empowered to Self-Care: A Photovoice Study in Patients With Heart Failure. *J Transcult Nurs* 2018;**29**:410–9. doi:10.1177/1043659617745138

31 GLEESON K. Polytextual Thematic Analysis for visual data – pinning down the analytic. In: *Visual Methods in Psychology*. Routledge 2011.

32 Han CS, Oliffe JL. Photovoice in mental illness research: A review and recommendations. *Health (N Y)* 2016;**20**:110–26. doi:10.1177/1363459314567790

33 Tsang KK. Photovoice Data Analysis: Critical Approach, Phenomenological Approach, and Beyond. *Beijing Int Rev Educ* 2020;**2**:136–52. doi:10.1163/25902539-00201009

34 Experience-based investigation and codesign of approaches to prevent and reduce Mental Health Act use (CO-PACT): a protocol | BMJ Open. https://bmjopen.bmj.com/content/12/2/e060086.abstract (accessed 25 Feb 2022).

35 Sutton-Brown CA. Photovoice: A Methodological Guide. *Photogr Cult* 2014;**7**:169–85. doi:10.2752/175145214X13999922103165

36 Remembering One's Stay in Hospital: A Study in Photography, Recovery and Forgetting - Alan Radley, Diane Taylor, 2003. https://journals.sagepub.com/doi/abs/10.1177/1363459303007002872?casa_token=Ld7KzjvM3hYAAAAA:U7TJUWgeZddjF96WvPeiw9y0qJdX-h8a3DwmrLY1r-laaeY_dd7QOaOYbvqXSrvjnJ51mEf__K_2uQ (accessed 26 Feb 2022).

For peer review only

Acknowledgements

For peer review only

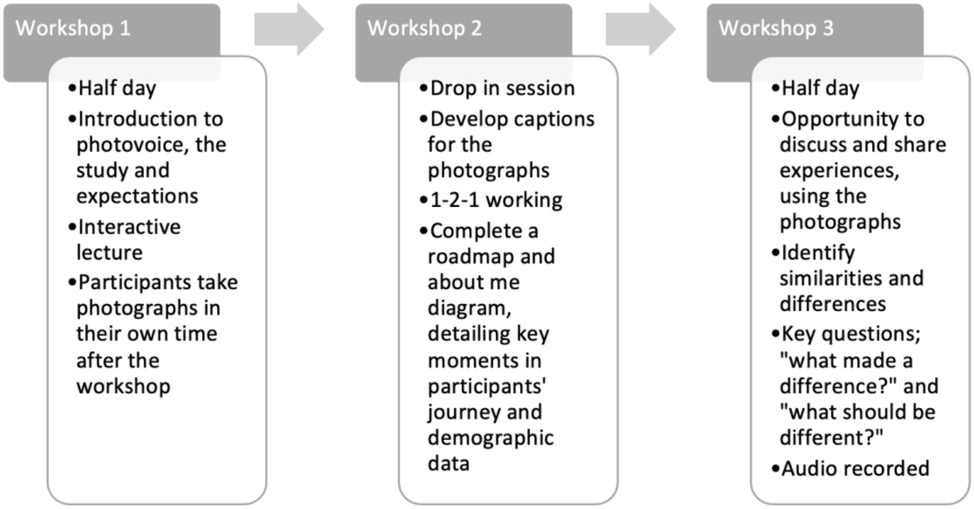

274x145mm (144 x 144 DPI)

BMJ Open

Analysing multi modal data that has been collected using photovoice as a research method

Journal:	*BMJ Open*
Manuscript ID	bmjopen-2022-068289.R2
Article Type:	Communication
Date Submitted by the Author:	27-Feb-2023
Complete List of Authors:	Mooney, Roisin; University of Oxford, Psychiatry<br>Bhui , Kamaldeep; University of Oxford,<br>Project Team , Co-Pact ; Oxford University
**Primary Subject Heading**:	Health services research
Secondary Subject Heading:	Qualitative research, Mental health, Communication
Keywords:	QUALITATIVE RESEARCH, MENTAL HEALTH, PSYCHIATRY

SCHOLARONE™
Manuscripts

I, the Submitting Author has the right to grant and does grant on behalf of all authors of the Work (as defined in the below author licence), an exclusive licence and/or a non-exclusive licence for contributions from authors who are: i) UK Crown employees; ii) where BMJ has agreed a CC-BY licence shall apply, and/or iii) in accordance with the terms applicable for US Federal Government officers or employees acting as part of their official duties; on a worldwide, perpetual, irrevocable, royalty-free basis to BMJ Publishing Group Ltd ("BMJ") its licensees and where the relevant Journal is co-owned by BMJ to the co-owners of the Journal, to publish the Work in BMJ Open and any other BMJ products and to exploit all rights, as set out in our licence.

The Submitting Author accepts and understands that any supply made under these terms is made by BMJ to the Submitting Author unless you are acting as an employee on behalf of your employer or a postgraduate student of an affiliated institution which is paying any applicable article publishing charge ("APC") for Open Access articles. Where the Submitting Author wishes to make the Work available on an Open Access basis (and intends to pay the relevant APC), the terms of reuse of such Open Access shall be governed by a Creative Commons licence – details of these licences and which Creative Commons licence will apply to this Work are set out in our licence referred to above.

Other than as permitted in any relevant BMJ Author's Self Archiving Policies, I confirm this Work has not been accepted for publication elsewhere, is not being considered for publication elsewhere and does not duplicate material already published. I confirm all authors consent to publication of this Work and authorise the granting of this licence.

For peer review only

Analysing multi modal data that has been collected using photovoice as a research method

Dr Roisin Mooney CHiMES Collaborative, Dept of Psychiatry, University of Oxford
Prof. Kamaldeep Bhui, CHiMES Collaborative, Dept of Psychiatry, Nuffield Department of Primary Health Care Sciences, Wadham College, University of Oxford. Hon Consultant East London and Oxford NHS Foundation Trusts, WPA Collaborating Centre, Oxford. And Co-Pact Project Team.

Co-Pact Team; Doreen Joseph (Doreen.Joseph@psych.ox.ac.uk), Prof. Nusrat Husain (nusrat.husain@manchester.ac.uk), Prof. Rose McCabe (rose.mccabe@city.ac.uk), Prof. Paul McCrone (P.McCrone@greenwich.ac.uk), Dr Karen Newbigging (karen.newbigging@psych.ox.ac.uk), Prof. Raghu Ragahavan (rraghavan@dmu.ac.uk), Dr Clair Dempsey (clair.dempsey@psych.ox.ac.uk), Michelle Yeung (michelle.yeung@psych.ox.ac.uk)

Corresponding author: Dr Roisin Mooney
Roisin.mooney@psych.ox.ac.uk
CHiMES Collaborative
Deptartment of Psychiatry
University of Oxford
Warneford Hospital
Headington
Oxford
OX3 7JX

Abstract (121 words)

Creative arts practices can enhance the depth and quality of mental health research by capturing and foregrounding participants' lived experience. Creative methods are emotionally activating and promote multiple perspectives, tolerating ambiguities and uncertainties, which are shared and even celebrated. Methods such as photovoice use imagery to elucidate narratives that are not easily captured by more traditional interview based research techniques. However, the use of creative methods and participatory research remains

For peer review only

novel as there is little established guidance or consensus on how to navigate conceptual, practical, and analytical challenges. This paper considers these challenges, and puts forward practical and theory informed recommendations, illustrated by a study that uses photovoice methods to investigate ethnic inequalities in the use of the mental health act (Co-Pact) as a case study.

Photovoice as a research method (246 words)

The use of photography and visual images as a form of research (photovoice) is an increasingly popular way to gather experiences that are overlooked, hidden or difficult to articulate. The purpose is to give 'voice' to experiences of injustice and adversity in a psychologically safe way. This knowledge can be used to inform the design of more inclusive policies, public services, and practice [1].

Grounded in feminist theory and empowerment, photovoice was first developed as a method to engage communities in sharing their lived experiences in the early 1990s, and was used in areas of health education, community activism, and public services [2]. The growth of visual research methods may in part be attributable to the rise in contemporary visual culture [3]. In the last 30 years the use of photovoice has been adapted for many different contexts [1][4]. The approach is gaining traction as a qualitative research method that enables non-verbal, emotional, pre-conscious, and creatives processes to be activated, in turn enabling difficult experiences to be surfaced and explored. For these reasons this approach may be of particular use when working with children and young people [5], and people from marginalised populations [1].

Participation in photovoice has been demonstrated to empower participants [6], improve reflexivity [7], and promote a sense of personal accountability [8]. Creative methods generally, and photovoice specifically, can help to reduce power imbalances between researchers and research participants, by both parties interpreting the image and placing the participant's voice centre stage. This is especially important where there is a lack of trust, concerns about institutional harms, or lack of psychological safety (as is the case for ethnic minorities in contact with mental health services), and where stigma may also prevent openness.

Implementing photovoice (424 words)

For peer review only

Photovoice methods involve posing a question and asking people to represent their views or sentiments through photographs or photo-assignments, followed by a process of interpretation or photo-discussion. The methodology can be adapted in lots of different settings; photographs or images could be generated using cameras, making use of existing imagery, or even using drawings [9]. Data collection may happen individually or in groups, eliciting both visual (photographs) and narrative data (participants' voices). There is no convention as to the number of photo-assignments or photo-discussions, nor the duration of meetings and ways of assembling, analysing and presenting the resulting data. The data collection and analysis may consist of a linear approach by which participants, take photographs, interpret them and share them with each other, and/or with researchers. The assumption is that material is produced and unambiguously interpreted by the participants themselves, then presented for wider discussion to generate consensus. Alternatively, some researchers have adopted a more iterative (whereby the process develops in repeating each stage, or iteration) and multi-layered approach whereby a series of photo-assignments, and related discussions are held, with evolving meanings and re-appraisal of motivations, affects, attitudes, and emergent interpretations over time [10]. This permits a multitude of perspectives which may be diverse and contradictory even but represent valuable insights into lived experience and perspectives of importance to participants.

The photographs shared as part of the photovoice process provide a rich detailed insight as to how the participants experience, interact with and understand the world around them, enabling those viewing the images to see the world from the participants' perspective. Data captured in photographs goes beyond the visual description of the content of the photograph; for example, metaphors are often used to convey feelings or experiences that are difficult to put into words. Photographs may be interpreted subjectively and are likely therefore to give expression to aspects of experience that might not be recognised, acknowledged, or even shared in a group. There may be social norms to which individuals feel obliged to conform.

There are multiple levels of content, and hence, the analysis and interpretation must be appropriately nuanced. For example, the way a person chooses to compose the images, the subject matter, but also the way they engage with the task, the format (virtual or face to face) the timing, and types of camera used. All of these unspoken perspectives may emerge in and impact the interpretation and analysis. Furthermore, the form of picture development, black and white, sepia, or grained, and use of digital tools to modify photographs are all possible

For peer review only

now. Undertaking the exercise in groups with other service users or peer researchers can also provide a more supportive and facilitative environment where critical reflection of the experiences and the potential for new understandings emerge from the discussion.

Embedding rigor to maintain research integrity (308 words)

As the photovoice approach gains popularity in part attributable to the significant flexibility it offers, it is important to ensure that the underpinning theory is understood and considered in both collecting data and the consequent analytic approach adopted [11]. The potential for experiential data to inform policy is progressive, and likely to be an important way of ensuring inclusive research that fully represents marginalised perspectives. However, the stages of moving through recruitment of participants, data collection, data storage, coding the data, extracting information, analysis and dissemination are not well established in mental health research and there are no standardised reporting frameworks.

As creative processes are not reliant on language, nor limited by time-pressured interviews, they are more supportive of enabling participants to gradually share fragile, difficult to verbalise, overlooked, or hidden experiences at their own pace. It is important to ensure clarity of process and document decisions, so that the contributions of participants are fully represented and are not lost, diluted or redefined in the process of data collection and analysis. Furthermore, the differences between facilitating creative processes for community engagement versus for research should be clearly acknowledged. This incorporates gaining and documenting the relevant ethics approvals, ensuring appropriate support in place for participants, having discussions about how any information participants provide could be used and ensuring that participants are aware of their right to withdraw from the research at any time. Whilst it is common in research for participant contributions to be confidential and anonymised, it is important that should participants want to be credited alongside their creative contributions during the dissemination of findings, that this should be considered and facilitated.

The interpretation process can involve other people which can lead to the generation of additional perspectives and layers of meaning. For example, in group discussions, viewing photographs taken by some participants may facilitate others to verbalise their own experiences. Accurate documentation and ensuring that all the data is considered

For peer review only

systematically using an analytic framework as opposed to sharing preliminary emerging narratives or summaries, further differentiates research from public engagement.

Analytic challenges arising from photovoice (1185 words)

Wang and Burris wrote “photographs are easy to gather but difficult to analyse and summarise because they yield an abundance of complex data that can be difficult to digest” (p375) [12]. Furthermore, it isn’t only the photographs that have been taken that are presented and discussed by participants in photo-discussions, but also the photographs that they were unable to take, or chose not to take [13]. This notion of complexity is compounded by the lack of standardisation regarding how photovoice is implemented. Participatory processes require flexibility yet guidance for researchers is lacking on different approaches, and helpful approaches. The number of photographs being considered, the number of interactions between participants and researchers, and the number of interactions between different participants can vary greatly, all of which have an impact on the volume and quality of the data collected, and on the narrative reflections on the images. Yet it is unknown what the optimal volume is, and what could indicate high quality photovoice data. These indicators may also vary according to the purpose and structure that photovoice is being conducted within. For example, shorter captions to provide context to images, may be appropriate if there are multiple iterative group discussions. Whereas longer captions may be preferable if there are fewer interactions or discussions between the participants themselves or with the researcher.

Many traditions in qualitative psychological research have been generated based on oral or written data. Whilst the interpretation of speech or text can vary according to its intention, and the experiences of the person interpreting it, most words tend to have a clear and universally acknowledged meaning. Conversely interpreting photographs is subjective. Photographs may have multiple meanings and many different interpretations. Generally photographs have a public meaning (universally recognisable elements) and a private meaning (partial objects in the pictures that have an emotional origin and are difficult to express in words) [14][15]. It is important to understand how participants assign meaning to their images and create a space that enables them to communicate their private meaning to both the researchers and other participants. This process also permits the participant, the owner of the photograph, to retain control about what is communicated and what is silenced. The photos act as an elicitation tool, to help reflection, recall, and verbalisation of

For peer review only

unexpressed views[16] . Fostering collaboration between researchers and participants may minimise power imbalances, and elicit new, deeper understanding as opposed to the potentially rehearsed narratives that often manifest in traditional forms of data collection such as interviews and questionnaires. [9]

The analysis of photovoice data typically includes inductive forms of analysis such as interpretative phenomenological analysis (IPA)[17][18][19], grounded theory [20][21][22] and thematic analysis [23][20][24]. Inductive approaches use the data as a starting point for the analysis as opposed to applying a deductive framework based on existing knowledge, however "we cannot conduct research without making assumptions" (p9) [25]. There are assumptions about how knowledge is created and communicated that underpin qualitative forms of analysis. It is important to understand how these assumptions manifest and the context within which the data was collected when deciding which form of analysis is appropriate. A core component of photovoice is the critical consciousness generated by the discussion of photographs [26], yet many of the psychological underpinnings in qualitative research are developed from a preoccupation with how an individual constructs and understands the world around them.

IPA aims to provide an in depth understanding of not only what an experience was like for participants but also how they made sense of it. Therefore, traditionally IPA involves two stages of interpretation; how the participants interpret their experiences, and how researchers interpret participants interpreting their experience [27]. The second stage risks diluting the voice of the participant. When adapting this approach for photovoice data some researchers add a data verification step [18]; others have analysed individual interviews about the photographs participants have taken using IPA, then looked for themes across these interviews [28] [17], or analysed transcripts of group discussions using IPA [19]. Whilst IPA may appear well suited for photovoice data owing to the ability view the world through the lens of the participant when examining photographs with the meaning ascribed to them by the participants themselves [29], this approach may not be well suited to large volumes of data that contains multiple layers (such as the captions for the individual photographs and the group discussions).

Grounded theory method is an iterative form of analysis that seeks to develop new explanations concerning basic social processes [30]. This form of analysis may be useful

For peer review only

when there are multiple photovoice workshops or photo-assignment sessions, to enable the iterative development of a new theory grounded in lived experience [31]. The assumptions underpinning grounded theory also speak to participatory action research (PAR) methodology that collaborates with (rather than studies) people to develop new ways of seeing the world [32]. In practice this method may entail the development of a code book that can be applied to both visual and narrative data, alongside constant comparison and data verification with participants to ensure authenticity of the findings [21]. Interviews or focus groups may be conducted to elicit the meaning associated with the photographs, and the resulting transcripts analysed using grounded theory method [22]. This approach is particularly useful where there is capacity to hold a series of photovoice workshops or photo assignments and allow an iterative process.

Thematic analysis is described by Braun and Clarke as a method for identifying, analysing and reporting patterns within data [33]. The core aim of identifying patterns also renders this method suitable for both visual and narrative data. Thematic analysis has gained popularity over the last 15 years owing to its flexibility and accessibility for interdisciplinary researchers. The seven steps, transcription, familiarisation, coding, searching for themes, reviewing themes, defining themes, and finalising themes; provide a rigorous framework that can be applied to large volumes of data in different contexts. Thematic analysis is commonly used within the psychological research approach where photovoice interviews have been employed with individuals [24][34][23]. When combining PAR and psychological research, the thematic approach is well suited to facilitating participants to collaborate in the analysis, by assisting in the identification of patterns across their data. Poly textual thematic analysis, is a further development of thematic analysis principles to incorporate visual data [35]. This approach allows researchers to analyse how photographs are interpreted by participants, as opposed to being analysed and interpreted by researchers. This approach appears optimal to ensure that photographs and narratives are never analysed independently of each other [10].

A qualitative scoping review of how photovoice was used in mental illness research found 9 studies, of which 7 analysed primary data [36]. The PAR approach and collaboration with the communities was central to primary studies. Grounded theory was the most cited analytical framework, however processes relating to the thematic analysis were often described in the absence of an explicitly stated analytical framework. Most studies involved participants in the analysis stage, but the majority were (n=7) were still perceived to be researcher-centric [36].

For peer review only

Within psychological research it has been proposed that the researchers and participants should analyse or interpret photographs independently, then compare their findings to generate holistic explanations and theorise phenomenon [37]. Of the 9 studies only 2 included formal analysis of the photographs.

Co-Pact: A Case Study (784 words)

Co-PACT is an NIHR funded study that seeks to combine photovoice and experience based co design [38] with the aim of reducing the ethnic inequalities experienced by those detained under the Mental Health Act (MHA). The use of photovoice to elucidate new perspectives and amplify the voices of marginalised populations in a creative and accessible manner renders it an optimal methodology to inform experience based co design meetings [39]. The intention of this study is to have an impact at a national policy level, by informing the current reform of the MHA. Photovoice data is being collected across eight NHS trusts from service users who have experience of being detained under the MHA in the last 48 months. Recruitment for these workshops has been purposive to ensure that those from marginalised backgrounds are facilitated to engage with the research process and to ensure that a range of difference section types, demographics and experiences are captured.

We have linked photovoice to experience based co-design to ensure service users themselves (working alongside other stakeholders) will have the power or ability to change an intrinsically complex system, that has resulted in their unequal treatment. We want to ensure that we allow for their authentic and undiluted voice to be heard in spaces where those who have the power to change the system can not only hear it but also engage with it. When discussing the photographs that they provide participants critically reflect on their own situation and produce materials that can be used to plan, inform and motivate change [40]. It is important for our analysis to capture the critical consciousness that results from the group dynamics (not solely analysis of the visual data and associated captions), to inform

For peer review only

experience based co-design workshops which will aim to elicit changes in local systems, and consequently inform national policy.

Our approach to photovoice consists of a series of three workshops (figure. 1), facilitated by researchers, delivered face to face or online according to participant preference. The first workshop addresses many ethical issues relating to the use of photovoice in research, and is detailed in our published protocol [41]. For example, participants were instructed not to include images of people who could be identified as they haven't provided consent to be a part of the study. We also work with participants in a continual collaborative way, reaffirming consent at each stage, including dissemination to ensure that they are comfortable and confident in how their contributions are being used.

[insert figure 1]

We have had many interdisciplinary workshops and discussions (inclusive of people who have experienced detention under the MHA) to refine our analytic approach to the photovoice data, whilst being cognisant of the tensions and challenges with the complexity, volume and depth of the data we have collected. In recognition that one of the aims of the study is to foreground lived experience, it is important to acknowledge that the analysis of the photographs begins with the participant's own interpretation of their images, developing captions and assigning meaning to their image. A second stage of analysis has also occurred with participants discussing their images in the third workshop and beginning to consider similarities and differences in their experiences, identifying patterns across the data. Based on approaches taken by other researchers working with visual data, and the principles of poly textual thematic analysis [35] we propose the following steps (table 1);

Table 1: Stages of analysis for multi-modal data for the Co-PACT study

Step 1- Understanding the sample	Extract relevant demographic information into an excel sheet (Sheet 1)<br>Initial extraction could include;<br>• Age,<br>• Gender,<br>• Ethnicity,<br>• Number of times sectioned,

For peer review only

	• Most recent section type and Diagnosis.<br><br>Fields may be added as patterns in the data are recognised, for example; Childhood trauma, age of onset and physical co-morbidities.
Step 2 – Organise the data	Organise data into a second excel spreadsheet (Sheet 2) containing the following headings;<br>• Image ID,<br>• Participant's caption,<br>• Image,<br>• Alternative Text Description (this will be generated by the research team, and will be a very basic narrative description, to assist the organisation of data),<br>• Mentions in transcript from the third workshop discussion,<br>• Similar Images and Participant Demographics.<br><br>This will enable a framework analysis of the data and a way to organise the data according to different variables. Researchers should keep a log book and note any observations they make during the process, such as how a group of images look together, and feelings or messages that are conveyed or noticed.
Step 3 – Individual Case Analysis	Examine the images and captions for each participant, consider similarities and themes within the images and the overall narrative that they are sharing.<br><br>Code both the visual content of the images and the captions relating to the image, as well as noting any observations about the group of images together. A code book (sheet 3) can be used to organise which participants have endorsed particular codes in the data they have contributed.
Step 4 – Exploring interactions	Go through the transcripts from the group discussions where participants discuss their images as a group, these should read as photo essays with the photographs added to the transcripts in the relevant places. Code any relevant information. Owing to the volume of data it may be preferable to

For peer review only

	use qualitative analysis software to code transcripts. We would recommend MAXQDA.<br><br>Extract any references to the images and add these to sheet 2, this should include any elements where multiple participants are discussing the same image.
Step 5 – Combining the individual and group analyses	Incorporate information from the transcripts (codes from step 4) to the existing case based analysis (Sheet 3). For a multi – site study, steps 2-5 should be done at a site level to allow an understanding of how the local context may impact the shared experiences.
Step 6- Theme generation	Review Sheet 2 (the data) and Sheet 3 (the codes from the images and transcripts) for all sites, alongside people with lived experience, to consider and develop how codes may be grouped together to develop higher order themes.<br><br>It is important that within this process, if there is a voice or experience that doesn't fit the dominant narrative that this isn't disregarded or diluted in the process. We should seek to understand the exceptions, and the potential reasons for these differences as well as the main themes.
Step 7 – Theme iteration	Review higher order themes, and the image/caption combinations within those, alongside field notes from the workshops as well as group notes generated from the discussion. This is to ensure that there aren't elements of the group discussion that were important to the participants who were present in the room that are missed.<br><br>The first step, in understanding the sample and collating the demographic information should be acknowledged here to provide context to the data being discussed.
Step 8 – Dissemination	Ensure that the outputs are used to motivate change, this should entail activities beyond publishing lay summaries and academic journal articles, such as presenting themes at co-design meetings and hosting public exhibitions of the images in collaboration with the participants.

For peer review only

When analysing photovoice data it is also important to remember the potential therapeutic impact on the participants from taking part in these workshops, and how this impacts the information they are sharing. The creative process, may have an impact on how the participants themselves relate to the experiences they are sharing, offering new insights as opposed to a rehearsed narrative. For marginalised communities, photovoice may be a more accessible way to part take in research, thus it is possible that the participants are very research naieve, not used to sharing their experiences and find benefit in participation.

We propose that the following questions are considered prior to analysis in future photovoice studies;

- What does the data consist of?
    - How many interactions are there between the participant and the research team?
    - How many images are there per participant?
    - How detailed is the accompanying narrative?
- What is the relationship between the participants and those who facilitated the workshops?
- Is there a group narrative, individual narrative or combination of both?
- Who is analysing the data?
- What is the analysis informing? For what purpose is the study being conducted?

Methodological advances in this area will only be possible if we continue to innovate, document and feedback our experiences of analysing visual and narrative data in combination. The development of creative, participatory methodologies such as photovoice are needed to further enable marginalised populations to contribute meaningfully towards research, and inform future innovations in health care.

Conclusion (98 words)

Photovoice is a creative way of enabling participants to share difficult experiences. Initially developed for the purpose of engaging communities and motivating social justice, there are a wide range of purposes for which photovoice has now been adopted. Researchers have used photovoice to elicit the narratives of marginalised populations whose voices are often

For peer review only

silenced. The flexibility in how photovoice is implemented renders numerous possibilities and challenges when analysing the resulting data. For researchers it is important to be transparent about the steps taken when processing the data that participants have generated and to share how decisions were informed.

For peer review only

Authors contributions:

RM drafted the initial manuscript that was revised by KB, and then continual revisions made collaboratively.

The content of this article reflects conversations held by the Co-Pact team, a project for which KB and RM are Co-PIs.

Funding Statement

This study/project is funded by the National Institute for Health Research (NIHR) Policy Research Programme (NIHR201704). The views expressed are those of the authors and not necessarily those of the NIHR or the Department of Health and Social Care.

Competing interests' statement

Nothing to declare

Figure caption

Page 8 - Figure 1: The photovoice workshop process adopted in Co-pact

For peer review only

1 Halvorsrud K, Rhodes J, Webster GM, *et al.* Photovoice as a promising public engagement approach: capturing and communicating ethnic minority people's lived experiences of severe mental illness and its treatment. *BMJ Open Qual* 2019;**8**:e000665. doi:10.1136/bmjoq-2019-000665

2 Wang C, Burris MA. Empowerment through Photo Novella: Portraits of Participation. *Health Educ Q* 1994;**21**:171–86. doi:10.1177/109019819402100204

3 Rose G. On the Relation between 'Visual Research Methods' and Contemporary Visual Culture. *Sociol Rev* 2014;**62**:24–46. doi:10.1111/1467-954X.12109

4 Halvorsrud K, Eylem O, Mooney R, *et al.* Identifying evidence of the effectiveness of photovoice: a systematic review and meta-analysis of the international healthcare literature. *J Public Health* Published Online First: 5 April 2021.https://doi.org/10.1093/pubmed/fdab074 (accessed 6 Sep 2021).

5 Abma TA, Schrijver J. 'Are we famous or something?' Participatory Health Research with children using photovoice. *Educ Action Res* 2020;**28**:405–26. doi:10.1080/09650792.2019.1627229

6 Budig K, Diez J, Conde P, *et al.* Photovoice and empowerment: evaluating the transformative potential of a participatory action research project. *BMC Public Health* 2018;**18**:432. doi:10.1186/s12889-018-5335-7

7 Hodgetts D, Chamberlain K, Groot S. 20 Reflections on the visual in community research and action. *Vis Methods Psychol Using Interpret Images Qual Res* 2012;:299.

8 Carlson ED, Engebretson J, Chamberlain RM. Photovoice as a Social Process of Critical Consciousness. *Qual Health Res* 2006;**16**:836–52. doi:10.1177/1049732306287525

9 Guillemin M, Drew S. Questions of process in participant-generated visual methodologies. *Vis Stud* 2010;**25**:175–88. doi:10.1080/1472586X.2010.502676

10 Törnbom K, Lundälv J, Palstam A, *et al.* "My life after stroke through a camera lens"- A photovoice study on participation in Sweden. *PLOS ONE* 2019;**14**:e0222099. doi:10.1371/journal.pone.0222099

11 Liebenberg L. Thinking Critically About Photovoice: Achieving Empowerment and Social Change. *Int J Qual Methods* 2018;**17**:1609406918757631. doi:10.1177/1609406918757631

12 Wang C, Burris MA. Photovoice: concept, methodology, and use for participatory needs assessment. *Health Educ Behav Off Publ Soc Public Health Educ* 1997;**24**:369–87. doi:10.1177/109019819702400309

13 Hodgetts D, Chamberlain K, Radley A. Considering Photographs Never Taken During Photo-production Projects. *Qual Res Psychol* 2007;**4**:263–80. doi:10.1080/14780880701583181

14 Rania N, Migliorini L, Rebora S, *et al.* Photovoice and Interpretation of Pictures in a Group Discussion: A Community Psychology Approach. *Qual Res Psychol* 2015;**12**:382–96. doi:10.1080/14780887.2015.1019597

For peer review only - http://bmjopen.bmj.com/site/about/guidelines.xhtml

15 Warren S. Photography and voice in critical qualitative management research. *Account Audit Account J* 2005;**18**:861–82. doi:10.1108/09513570510627748

16 Nykiforuk CIJ. Engaging patients in research using photovoice methodology. *CMAJ* 2021;**193**:E1050–1. doi:10.1503/cmaj.210963

17 annadadswell A. Interpretative phenomenological analysis and photovoice methods in participatory therapeutic contexts. ARU Particip. Res. 2021.https://aruparticipatoryresearch.wordpress.com/2021/03/25/interpretative-phenomenological-analysis-and-photovoice-methods-in-participatory-therapeutic-contexts/ (accessed 25 Feb 2022).

18 Tanhan A, Strack RW. Online photovoice to explore and advocate for Muslim biopsychosocial spiritual wellbeing and issues: Ecological systems theory and ally development. *Curr Psychol* 2020;**39**:2010–25. doi:10.1007/s12144-020-00692-6

19 Cosgrove D, Bozlak C, Reid P. Service Barriers for Gender Nonbinary Young Adults: Using Photovoice to Understand Support and Stigma. *Affilia* 2021;**36**:220–39. doi:10.1177/0886109920944535

20 Latz AO, Mulvihill TM. *Photovoice Research in Education and Beyond: A Practical Guide from Theory to Exhibition*. New York: : Routledge 2017. doi:10.4324/9781315724089

21 Cabassa LJ, Nicasio A, Whitley R. Picturing recovery: a photovoice exploration of recovery dimensions among people with serious mental illness. *Psychiatr Serv Wash DC* 2013;**64**:837–42. doi:10.1176/appi.ps.201200503

22 Santos MAF, Lopes MAP, Botelho MAR. Photovoice as a Method of Data Collection in the Study of Motherhood Over the Age of 35: The Power of Images. In: Costa AP, Reis LP, Souza FN de, *et al.*, eds. *Computer Supported Qualitative Research*. Cham: : Springer International Publishing 2018. 316–26. doi:10.1007/978-3-319-61121-1_27

23 Glaw X, Inder K, Kable A, *et al.* Visual Methodologies in Qualitative Research: Autophotography and Photo Elicitation Applied to Mental Health Research. *Int J Qual Methods* 2017;**16**:1609406917748215. doi:10.1177/1609406917748215

24 Edwards LB, Greeff LE. A descriptive qualitative study of childhood cancer challenges in South Africa: Thematic analysis of 68 photovoice contributions. *South Afr J Oncol* 2017;**1**:8. doi:10.4102/sajo.v1i0.14

25 Rohleder P, Lyons AC. *Qualitative Research in Clinical and Health Psychology*. Macmillan International Higher Education 2014.

26 Freire P. Pedagogy of the Oppressed. 1968. *Trans Myra Bergman Ramos N Y Herder* 1972.

27 Smith JA. Evaluating the contribution of interpretative phenomenological analysis. *Health Psychol Rev* 2011;**5**:9–27. doi:10.1080/17437199.2010.510659

28 Keating F. Black men's conversations about mental health through photos. *Qual Soc Work* 2021;**20**:755–72. doi:10.1177/1473325020922293

For peer review only

29 Brunsden V, Goatcher J. Reconfiguring Photovoice for Psychological Research. *Ir J Psychol* 2007;**28**:43–52. doi:10.1080/03033910.2007.10446247

30 Charmaz K. Reconstructing grounded theory. *SAGE Handb Soc Res Methods* 2008;:461–78.

31 Freedman DA, Pitner RO, Powers MCF, *et al.* Using Photovoice to Develop a Grounded Theory of Socio-Environmental Attributes Influencing the Health of Community Environments. *Br J Soc Work* 2014;**44**:1301–21. doi:10.1093/bjsw/bcs173

32 Bradbury H, Reason P. Action Research: An Opportunity for Revitalizing Research Purpose and Practices. *Qual Soc Work* 2003;**2**:155–75. doi:10.1177/1473325003002002003

33 Using thematic analysis in psychology: Qualitative Research in Psychology: Vol 3, No 2. https://www.tandfonline.com/doi/abs/10.1191/1478088706QP063OA (accessed 25 Feb 2022).

34 Chew HSJ, Lopez V. Empowered to Self-Care: A Photovoice Study in Patients With Heart Failure. *J Transcult Nurs* 2018;**29**:410–9. doi:10.1177/1043659617745138

35 GLEESON K. Polytextual Thematic Analysis for visual data – pinning down the analytic. In: *Visual Methods in Psychology*. Routledge 2011.

36 Han CS, Oliffe JL. Photovoice in mental illness research: A review and recommendations. *Health (N Y)* 2016;**20**:110–26. doi:10.1177/1363459314567790

37 Tsang KK. Photovoice Data Analysis: Critical Approach, Phenomenological Approach, and Beyond. *Beijing Int Rev Educ* 2020;**2**:136–52. doi:10.1163/25902539-00201009

38 Experience-based investigation and codesign of approaches to prevent and reduce Mental Health Act use (CO-PACT): a protocol | BMJ Open. https://bmjopen.bmj.com/content/12/2/e060086.abstract (accessed 25 Feb 2022).

39 Sutton-Brown CA. Photovoice: A Methodological Guide. *Photogr Cult* 2014;**7**:169–85. doi:10.2752/175145214X13999922103165

40 Remembering One's Stay in Hospital: A Study in Photography, Recovery and Forgetting - Alan Radley, Diane Taylor, 2003. https://journals.sagepub.com/doi/abs/10.1177/1363459303007002872?casa_token=Ld7KzjvM3hYAAAAA:U7TJUWgeZddjF96WvPeiw9y0qJdX-h8a3DwmrLY1r-laaeY_dd7QOaOYbvqXSrvjnJ51mEf__K_2uQ (accessed 26 Feb 2022).

41 Mooney R, Newbigging K, McCabe R, *et al.* Experience-based investigation and codesign of approaches to prevent and reduce Mental Health Act use (CO-PACT): a protocol. *BMJ Open* 2022;**12**:e060086. doi:10.1136/bmjopen-2021-060086

For peer review only

Acknowledgements

For peer review only

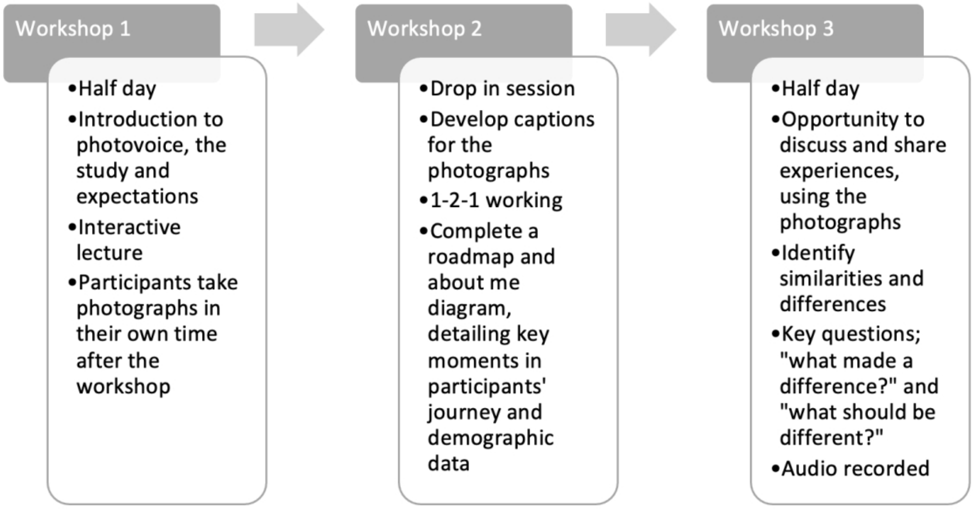

A brief description of content covered in a series of three photovoice workshops, undertaken with people who had experience of being detained under the Mental Health Act, conducted as part of the Co-PACT study.

274x145mm (144 x 144 DPI)

BMJ Open

Analysing multi modal data that has been collected using photovoice as a research method

Journal:	*BMJ Open*
Manuscript ID	bmjopen-2022-068289.R3
Article Type:	Communication
Date Submitted by the Author:	28-Mar-2023
Complete List of Authors:	Mooney, Roisin; University of Oxford, Psychiatry<br>Bhui , Kamaldeep; University of Oxford,<br>Project Team , Co-Pact ; Oxford University
**Primary Subject Heading**:	Health services research
Secondary Subject Heading:	Qualitative research, Mental health, Communication
Keywords:	QUALITATIVE RESEARCH, MENTAL HEALTH, PSYCHIATRY

SCHOLARONE™
Manuscripts

I, the Submitting Author has the right to grant and does grant on behalf of all authors of the Work (as defined in the below author licence), an exclusive licence and/or a non-exclusive licence for contributions from authors who are: i) UK Crown employees; ii) where BMJ has agreed a CC-BY licence shall apply, and/or iii) in accordance with the terms applicable for US Federal Government officers or employees acting as part of their official duties; on a worldwide, perpetual, irrevocable, royalty-free basis to BMJ Publishing Group Ltd ("BMJ") its licensees and where the relevant Journal is co-owned by BMJ to the co-owners of the Journal, to publish the Work in BMJ Open and any other BMJ products and to exploit all rights, as set out in our licence.

The Submitting Author accepts and understands that any supply made under these terms is made by BMJ to the Submitting Author unless you are acting as an employee on behalf of your employer or a postgraduate student of an affiliated institution which is paying any applicable article publishing charge ("APC") for Open Access articles. Where the Submitting Author wishes to make the Work available on an Open Access basis (and intends to pay the relevant APC), the terms of reuse of such Open Access shall be governed by a Creative Commons licence – details of these licences and which Creative Commons licence will apply to this Work are set out in our licence referred to above.

Other than as permitted in any relevant BMJ Author's Self Archiving Policies, I confirm this Work has not been accepted for publication elsewhere, is not being considered for publication elsewhere and does not duplicate material already published. I confirm all authors consent to publication of this Work and authorise the granting of this licence.

For peer review only

Analysing multi modal data that has been collected using photovoice as a research method

Dr Roisin Mooney[1] & Prof. Kam Bhui[1, 2, 3] , Co-Pact Project Team.

1. CHiMES Collaborative, Dept of Psychiatry, University of Oxford
2. Nuffield Department of Primary Care Health Sciences, Wadham College, University of Oxford, Oxford, UK
3. Queen Mary University London Global Policy Institute, London, UK

Co-Pact Project Team; Doreen Joseph (Doreen.Joseph@psych.ox.ac.uk), Prof. Nusrat Husain (nusrat.husain@manchester.ac.uk), Prof. Rose McCabe (rose.mccabe@city.ac.uk), Prof. Paul McCrone (P.McCrone@greenwich.ac.uk), Dr Karen Newbigging (karen.newbigging@psych.ox.ac.uk), Prof. Raghu Ragahavan (rraghavan@dmu.ac.uk), Dr Clair Dempsey (clair.dempsey@psych.ox.ac.uk), Michelle Yeung (michelle.yeung@psych.ox.ac.uk)

Corresponding author: Dr Roisin Mooney
Roisin.mooney@psych.ox.ac.uk
CHiMES Collaborative
Deptartment of Psychiatry
University of Oxford
Warneford Hospital
Headington
Oxford
OX3 7JX

For peer review only

Abstract

Background: Creative arts practice can enhance the depth and quality of mental health research by capturing and foregrounding participants' lived experience. Creative methods are emotionally activating and promote multiple perspectives, tolerating ambiguities and uncertainties, which are shared and even celebrated.

Key arguments: Methods such as photovoice use imagery to elucidate narratives that are not easily captured by more traditional interview based research techniques. However, the use of creative methods and participatory research remains novel as there is little guidance of how to navigate conceptual, practical, and analytical challenges.

Conclusion: This paper considers these challenges, and puts forward practical and theory informed recommendations, using as study of photovoice methods for investigating ethnic inequalities in the use of the mental health act (Co-Pact) as a case study.

For peer review only

Photovoice as a research method

The use of photography and visual images as a form of research (photovoice) is an increasingly popular way to gather patient experiences that are overlooked, hidden or difficult to articulate. The purpose is to give 'voice' to experiences of injustice and adversity in a psychologically safe way. This knowledge can be used to inform the design of more inclusive policies, public services, and practice [1].

Grounded in feminist theory and empowerment, photovoice was first developed as a method to engage communities in sharing their lived experiences in the early 1990s, and was used in areas of health education, community activism, and public services [2]. In the last 30 years photovoice has been adapted for many different contexts [1][3]. Thus, the approach is gaining traction as a qualitative research method that enables non-verbal, emotional, pre-conscious, and creatives processes to be activated, in turn enabling difficult experiences to be surfaced and explored. Participation in photovoice has been demonstrated to empower participants [4], improve reflexivity [5], and promote a sense of personal accountability [6]. Creative methods generally, and photovoice specifically, can help to reduce power imbalances between researchers and research participants, by both parties interpreting the image and placing the participant's voice centre stage. This is especially important where there is a lack of trust, concerns about institutional harms, or lack of psychological safety (as is the case for ethnic minorities in contact with mental health services), and where stigma may also prevent openness.

Implementing photovoice

Photovoice methods involve posing a question and asking people to represent their views or sentiments through photographs or photo-assignments, followed by a process of interpretation or photo-discussion. This can be done individually or in groups, eliciting both visual (photographs) and narrative data (participants' voices). There is no convention as to the number of photo-assignments or photo-discussions, nor the duration of meetings and ways of assembling, analysing and presenting the resulting data. The data collection and analysis may consist of a linear approach by which participants, take photographs, interpret them and share them with each other, and/or with researchers. The assumption is that material is produced and unambiguously interpreted by the participants themselves, then presented for wider discussion to generate consensus. Alternatively, some researchers have adopted a more iterative and multi-layered approach whereby a series of photo-assignments, and related

For peer review only

discussions are held, with evolving meanings and re-appraisal of motivations, affects, attitudes, and emergent interpretations over time [7]. This permits a multitude of perspectives which may be diverse and contradictory even, but represent valuable insights into lived experience and perspectives of importance to participants.

The photographs shared as part of the photovoice process provide a rich detailed insight as to how the participants experience, interact with and understand the world around them, enabling those viewing the images to see the world from the participants' perspective. Data captured in photographs goes beyond the visual description of the content of the photograph; for example, metaphors are often used to convey feelings or experiences that are difficult to put into words. Photographs may be interpreted subjectively and are likely therefore to give expression to aspects of experience that might not be recognised, acknowledged, or even shared in a group. There may be social norms to which individuals feel obliged to conform.

There are multiple levels of content, and hence, the analysis and interpretation must be appropriately nuanced. For example, the way a person chooses to compose the images, the subject matter, but also the way they engage with the task, the format (virtual or face to face) the timing, and types of camera used. All of these unspoken perspectives may emerge in and impact the interpretation and analysis. Furthermore, the form of picture development, black and white, sepia, or grained, and use of digital tools to modify photographs are all possible now. Undertaking the exercise in groups with other service users or peer researchers can also provide a more supportive and facilitative environment where critical reflection of the experiences and the potential for new understandings emerge from the discussion.

Embedding rigor to maintain research integrity

As the photovoice approach gains popularity in part attributable to the significant flexibility it offers, it is important to ensure that the underpinning theory is understood and considered in both collecting data and the consequent analytic approach adopted [8]. The potential for experiential data to inform policy is progressive, and likely to be an important way of ensuring inclusive research that fully represents marginalised perspectives. However, the stages of moving through recruitment of participants, data collection, data storage, coding the data, extracting information, analysis and dissemination are less well defined. This lack of guidance is helpful if the photograph is seen as an art product, subject to multiple interpretations and meaning-making as an essential attribute that engages and promotes

For peer review only

eudemonic satisfaction. Indeed, art in part seeks to resist reductive classifications and analysis and open the viewer and group of viewers to interact around shared and unshared experiences and interpretation.

As creative processes are not reliant on language, nor time-pressured interviews, and more supportive of staged elicitation of views, experiences that are fragile and difficult to verbalise, overlooked, or hidden most of the time can emerge and be deepened during the research. Visual and experience-near narratives are continually available to be used in a variety of contexts, such as being curated for public display, or synthesised with evidence from other research methods that, if used alone, risk losing insights from marginalised groups. It is important to ensure clarity of process and document decisions, so that the contributions of participants are fully represented and are not lost, diluted or redefined in the analytic process and summary statements.

The interpretation process can involve other people which can lead to the generation of additional perspectives and layers of meaning. Understanding these different layers is critically important in the analysis phase, but also to ensure authenticity in attributions of similar or different meanings.

Analytic challenges arising from photovoice

Wang and Burris wrote "photographs are easy to gather but difficult to analyse and summarise because they yield an abundance of complex data that can be difficult to digest" (p375) [9]. Furthermore, it isn't only the photographs that have been taken that are presented and discussed by participants in photo-discussions, but also the photographs that they were unable to take, or chose not to take [10]. This notion of complexity is compounded by the lack of standardisation regarding how photovoice is implemented. The number of photographs being considered, the number of interactions between participants and researchers, and the number of interactions between different participants can vary greatly, all of which have an impact on the volume and quality of the data collected. Yet it is unknown what the optimal volume is, and what could indicate high quality photovoice data. These indicators may also vary according to the purpose and structure that photovoice is being conducted within. For example, shorter captions to provide context to images, may be appropriate if there are multiple iterative group discussions. Whereas longer captions may be

For peer review only

preferable if there are fewer interactions or discussions between the participants themselves or with the researcher.

Many traditions in qualitative psychological research have been generated based on oral or written data. Whilst the interpretation of speech or text can vary according to its intention, and the experiences of the person interpreting it, most words tend to have a clear and universally acknowledged meaning. Conversely interpreting photographs is subjective. Photographs may have multiple meanings and many different interpretations. Generally photographs have a public meaning (universally recognisable elements) and a private meaning (partial objects in the pictures that have an emotional origin and are difficult to express in words) [11][12]. It is important to understand how participants assign meaning to their images and create a space that enables them to communicate their private meaning to both the researchers and other participants. This process also permits the participant, the owner of the photograph, to retain control about what is communicated and what is silenced. The photos act as an elicitation tool, to help reflection, recall, and verbalisation of unexpressed views. Fostering collaboration between researchers and participants may minimise power imbalances, and elicit new, deeper understanding as opposed to the potentially rehearsed narratives that often manifest in traditional forms of data collection such as interviews and questionnaires.

The analysis of photovoice data typically includes inductive forms of analysis such as interpretative phenomenological analysis (IPA)[13][14][15], grounded theory [16][17][18] and thematic analysis [19][16][20]. Inductive approaches use the data as a starting point for the analysis as opposed to applying a deductive framework based on existing knowledge, however "we cannot conduct research without making assumptions" (p9) [21]. There are assumptions about how knowledge is created and communicated that underpin qualitative forms of analysis. It is important to understand how these assumptions manifest and the context within which the data was collected when deciding which form of analysis is appropriate. A core component of photovoice is the critical consciousness generated by the discussion of photographs [22], yet many of the psychological underpinnings in qualitative research are developed from a preoccupation with how an individual constructs and understands the world around them.

For peer review only

IPA aims to provide an in depth understanding of not only what an experience was like for participants but also how they made sense of it. Therefore, traditionally IPA involves two stages of interpretation; how the participants interpret their experiences, and how researchers interpret participants interpreting their experience [23]. The second stage risks diluting the voice of the participant. When adapting this approach for photovoice data some researchers add a data verification step [14]; others have analysed individual interviews about the photographs participants have taken using IPA, then looked for themes across these interviews [24] [13], or analysed transcripts of group discussions using IPA [15]. Whilst IPA may appear well suited for photovoice data owing to the ability view the world through the lens of the participant when examining photographs with the meaning ascribed to them by the participants themselves [25], this approach may not be well suited to large volumes of data that contains multiple layers (such as the captions for the individual photographs and the group discussions).

Grounded theory method is an iterative form of analysis that seeks to develop new explanations concerning basic social processes [26]. This form of analysis may be useful when there are multiple photovoice workshops or photo-assignment sessions, to enable the iterative development of a new theory grounded in lived experience [27]. The assumptions underpinning grounded theory also speak to participatory action research (PAR) methodology that collaborates with (rather than studies) people to develop new ways of seeing the world [28]. In practice this method may entail the development of a code book that can be applied to both visual and narrative data, alongside constant comparison and data verification with participants to ensure authenticity of the findings [17]. Interviews or focus groups may be conducted to elicit the meaning associated with the photographs, and the resulting transcripts analysed using grounded theory method [18]. This approach is particularly useful where there is capacity to hold a series of photovoice workshops or photo assignments and allow an iterative process.

Thematic analysis is described by Braun and Clarke as a method for identifying, analysing and reporting patterns within data [29]. The core aim of identifying patterns also renders this method suitable for both visual and narrative data. Thematic analysis has gained popularity over the last 15 years owing to its flexibility and accessibility for interdisciplinary researchers. The seven steps, transcription, familiarisation, coding, searching for themes, reviewing themes, defining themes, and finalising themes; provide a rigorous framework that

For peer review only

can be applied to large volumes of data in different contexts. Thematic analysis is commonly used within the psychological research approach where photovoice interviews have been employed with individuals [20][30][19]. When combining PAR and psychological research, the thematic approach is well suited to facilitating participants to collaborate in the analysis, by assisting in the identification of patterns across their data. Poly textual thematic analysis, is a further development of thematic analysis principles to incorporate visual data [31]. This approach allows researchers to analyse how photographs are interpreted by participants, as opposed to being analysed and interpreted by researchers. This approach appears optimal to ensure that photographs and narratives are never analysed independently of each other [7].

A qualitative scoping review of how photovoice was used in mental illness research found 9 studies, of which 7 analysed primary data [32]. The PAR approach and collaboration with the communities was central to primary studies. Grounded theory was the most cited analytical framework, however processes relating to the thematic analysis were often described in the absence of an explicitly stated analytical framework. Most studies involved participants in the analysis stage, but the majority were (n=7) were still perceived to be researcher-centric [32]. Within psychological research it has been proposed that the researchers and participants should analyse or interpret photographs independently, then compare their findings to generate holistic explanations and theorise phenomenon [33]. Of the 9 studies only 2 included formal analysis of the photographs.

Co-Pact: A Case Study

Co-PACT is an NIHR funded study that seeks to combine photovoice and experience based co design [34] with the aim of reducing the ethnic inequalities experienced by those detained under the Mental Health Act (MHA). The use of photovoice to elucidate new perspectives and amplify the voices of marginalised populations in a creative and accessible manner renders it an optimal methodology to inform experience based co design meetings [35]. The intention of this study is to have an impact at a national policy level, by informing the current reform of the MHA. Photovoice data is being collected across eight NHS trusts from service users who have experience of being detained under the MHA in the last 48 months.

For peer review only

Recruitment for these workshops has been purposive to ensure that those from marginalised backgrounds are facilitated to engage with the research process and to ensure that a range of difference section types, demographics and experiences are captured.

We have linked photovoice to experience based co-design to ensure service users themselves (working alongside other stakeholders) will have the power or ability to change an intrinsically complex system, that has resulted in their unequal treatment. We want to ensure that we allow for their authentic and undiluted voice to be heard in spaces where those who have the power to change the system can not only hear it but also engage with it. When discussing the photographs that they provide participants critically reflect on their own situation and produce materials that can be used to plan, inform and motivate change [36]. It is important for our analysis to capture the critical consciousness that results from the group dynamics (not solely analysis of the visual data and associated captions), to inform experience based co-design workshops which will aim to elicit changes in local systems, and consequently inform national policy.

Our approach to photovoice consists of a series of three workshops (figure. 1), facilitated by researchers, delivered face to face or online according to participant preference.

[insert figure 1]

We have had many interdisciplinary workshops and discussions (inclusive of people who have experienced detention under the MHA) to refine our analytic approach to the photovoice data, whilst being cognisant of the tensions and challenges with the complexity, volume and depth of the data we have collected. In recognition that one of the aims of the study is to foreground lived experience, it is important to acknowledge that the analysis of the photographs begins with the participant's own interpretation of their images, developing captions and assigning meaning to their image. A second stage of analysis has also occurred with participants discussing their images in the third workshop and beginning to consider similarities and differences in their experiences, identifying patterns across the data. Based

For peer review only

on approaches taken by other researchers working with visual data, and the principles of poly textual thematic analysis [31] we propose the following steps (table 1);

Table 1: Stages of analysis for multi-modal data in Co-pact

Step 1- Understanding the sample	Extract demographic information from 'about me' diagrams and roadmaps into an excel sheet (Sheet 1) Initial extraction includes; Age, Gender, Ethnicity, Number of times sectioned, Most recent section type and Diagnosis. Fields may be added as patterns in the data are recognised, for example; Childhood trauma, age of onset and physical co-morbidities.
Step 2 – Organise the data	Organise data into a second excel spreadsheet (Sheet 2) containing the following headings; Image ID, Participant's caption, Image, Alternative Text Description (this will be generated by the research team, and will be a very basic narrative description, to assist the organisation of data), Mentions in transcript from the third workshop discussion, Similar Images and Participant Demographics. This will enable a framework analysis of the data and a way to organise the data according to different variables. Researchers should keep a log book and note any observations they make during the process, such as how a group of images look together, and feelings or messages that are conveyed or noticed.
Step 3 – Individual Case Analysis	Consider the images and captions for each participant, consider similarities and themes within the images and the overall narrative that they are sharing. Code both the visual content of the images and the captions relating to the image, as well as noting any observations about the group of images together.
Step 4 – Exploring interactions	Go through the transcripts from the group discussions where participants discuss their images as a group, these should read as photo essays with the photographs added to the transcripts in the relevant places. Extract any

For peer review only

	references to the images and add these to sheet 2, this should include any elements where multiple participants are discussing the same image.
Step 5 – Combining the individual and group analyses	Incorporate information from the transcripts to the existing case based analysis (Sheet 3). For a multi – site study, steps 2-5 should be done at a site level to allow an understanding of how the local context may impact the shared experiences.
Step 6- Theme generation	Review Sheet 2 (the data) and Sheet 3 (the codes) for all sites, alongside people with lived experience, to consider and develop how codes may be grouped together to develop higher order themes. It is important that within this process, if there is a voice or experience that doesn't fit the dominant narrative that this isn't disregarded or diluted in the process. We should seek to understand the exceptions, and the potential reasons for these differences as well as the main themes.
Step 7 – Theme iteration	Review higher order themes, and the image/caption combinations within those, alongside field notes from the workshops as well as group notes generated from the discussion. This is to ensure that there aren't elements of the group discussion that were important to the participants who were present in the room that are missed.
Step 8 – Dissemination	Ensure that the outputs are used to motivate change, this should entail activities beyond publishing lay summaries and academic journal articles, such as presenting themes at co-design meetings and hosting public exhibitions of the images in collaboration with the participants.

When analysing photovoice data it is also important to remember the potential therapeutic impact on the participants from taking part in these workshops, and how this impacts the information they are sharing. The creative process, may have an impact on how the participants themselves relate to the experiences they are sharing, offering new insights as opposed to a rehearsed narrative. For marginalised communities, photovoice may be a more accessible way to part take in research, thus it is possible that the participants are very research naïve, not used to sharing their experiences and find benefit in participation.

For peer review only

We propose that the following questions are considered prior to analysis in future photovoice studies;

- What does the data consist of?
    - How many interactions are there between the participant and the research team?
    - How many images are there per participant?
    - How detailed is the accompanying narrative?
- What is the relationship between the participants and those who facilitated the workshops?
- Is there a group narrative, individual narrative or combination of both?
- Who is analysing the data?
- What is the analysis informing? For what purpose is the study being conducted?

Methodological advances in this area will only be possible if we continue to innovate, document and feedback our experiences of analysing visual and narrative data in combination. The development of creative, participatory methodologies such as photovoice are needed to further enable marginalised populations to contribute meaningfully towards research, and inform future innovations in health care.

Conclusion

Photovoice is a creative way of enabling participants to share difficult experiences. Initially developed for the purpose of engaging communities and motivating social justice, there are a wide range of purposes for which photovoice has now been adopted. Researchers have used photovoice to elicit the narratives of marginalised populations whose voices are often silenced. The flexibility in how photovoice is implemented renders numerous possibilities and challenges when analysing the resulting data. For researchers it is important to be transparent about the steps taken when processing the data that participants have generated and to share how decisions were informed.

For peer review only

Authors contributions:

RM drafted the initial manuscript that was revised by KB, and then continual revisions made collaboratively.

The content of this article reflects conversations held by the Co-Pact team, a project for which KB and RM are Co-PIs.

Funding Statement

This study/project is funded by the National Institute for Health Research (NIHR) Policy Research Programme (NIHR201704). The views expressed are those of the authors and not necessarily those of the NIHR or the Department of Health and Social Care.

Ethics Approval Statement

Not Applicable

Competing interests' statement

Nothing to declare

Figure caption

Page 8 - Figure 1: The photovoice workshop process adopted in Co-pact

For peer review only

1 Halvorsrud K, Rhodes J, Webster GM, *et al.* Photovoice as a promising public engagement approach: capturing and communicating ethnic minority people's lived experiences of severe mental illness and its treatment. *BMJ Open Qual* 2019;**8**:e000665. doi:10.1136/bmjoq-2019-000665

2 Wang C, Burris MA. Empowerment through Photo Novella: Portraits of Participation. *Health Educ Q* 1994;**21**:171–86. doi:10.1177/109019819402100204

3 Halvorsrud K, Eylem O, Mooney R, *et al.* Identifying evidence of the effectiveness of photovoice: a systematic review and meta-analysis of the international healthcare literature. *J Public Health* Published Online First: 5 April 2021.https://doi.org/10.1093/pubmed/fdab074 (accessed 6 Sep 2021).

4 Budig K, Diez J, Conde P, *et al.* Photovoice and empowerment: evaluating the transformative potential of a participatory action research project. *BMC Public Health* 2018;**18**:432. doi:10.1186/s12889-018-5335-7

5 Hodgetts D, Chamberlain K, Groot S. 20 Reflections on the visual in community research and action. *Vis Methods Psychol Using Interpret Images Qual Res* 2012;:299.

6 Carlson ED, Engebretson J, Chamberlain RM. Photovoice as a Social Process of Critical Consciousness. *Qual Health Res* 2006;**16**:836–52. doi:10.1177/1049732306287525

7 Törnbom K, Lundälv J, Palstam A, *et al.* "My life after stroke through a camera lens"- A photovoice study on participation in Sweden. *PLOS ONE* 2019;**14**:e0222099. doi:10.1371/journal.pone.0222099

8 Liebenberg L. Thinking Critically About Photovoice: Achieving Empowerment and Social Change. *Int J Qual Methods* 2018;**17**:1609406918757631. doi:10.1177/1609406918757631

9 Wang C, Burris MA. Photovoice: concept, methodology, and use for participatory needs assessment. *Health Educ Behav Off Publ Soc Public Health Educ* 1997;**24**:369–87. doi:10.1177/109019819702400309

10 Hodgetts D, Chamberlain K, Radley A. Considering Photographs Never Taken During Photo-production Projects. *Qual Res Psychol* 2007;**4**:263–80. doi:10.1080/14780880701583181

11 Rania N, Migliorini L, Rebora S, *et al.* Photovoice and Interpretation of Pictures in a Group Discussion: A Community Psychology Approach. *Qual Res Psychol* 2015;**12**:382–96. doi:10.1080/14780887.2015.1019597

12 Warren S. Photography and voice in critical qualitative management research. *Account Audit Account J* 2005;**18**:861–82. doi:10.1108/09513570510627748

13 annadadswell A. Interpretative phenomenological analysis and photovoice methods in participatory therapeutic contexts. ARU Particip. Res. 2021.https://aruparticipatoryresearch.wordpress.com/2021/03/25/interpretative-phenomenological-analysis-and-photovoice-methods-in-participatory-therapeutic-contexts/ (accessed 25 Feb 2022).

For peer review only

14 Tanhan A, Strack RW. Online photovoice to explore and advocate for Muslim biopsychosocial spiritual wellbeing and issues: Ecological systems theory and ally development. *Curr Psychol* 2020;**39**:2010–25. doi:10.1007/s12144-020-00692-6

15 Cosgrove D, Bozlak C, Reid P. Service Barriers for Gender Nonbinary Young Adults: Using Photovoice to Understand Support and Stigma. *Affilia* 2021;**36**:220–39. doi:10.1177/0886109920944535

16 Latz AO, Mulvihill TM. *Photovoice Research in Education and Beyond: A Practical Guide from Theory to Exhibition*. New York: : Routledge 2017. doi:10.4324/9781315724089

17 Cabassa LJ, Nicasio A, Whitley R. Picturing recovery: a photovoice exploration of recovery dimensions among people with serious mental illness. *Psychiatr Serv Wash DC* 2013;**64**:837–42. doi:10.1176/appi.ps.201200503

18 Santos MAF, Lopes MAP, Botelho MAR. Photovoice as a Method of Data Collection in the Study of Motherhood Over the Age of 35: The Power of Images. In: Costa AP, Reis LP, Souza FN de, *et al.*, eds. *Computer Supported Qualitative Research*. Cham: : Springer International Publishing 2018. 316–26. doi:10.1007/978-3-319-61121-1_27

19 Glaw X, Inder K, Kable A, *et al.* Visual Methodologies in Qualitative Research: Autophotography and Photo Elicitation Applied to Mental Health Research. *Int J Qual Methods* 2017;**16**:1609406917748215. doi:10.1177/1609406917748215

20 Edwards LB, Greeff LE. A descriptive qualitative study of childhood cancer challenges in South Africa: Thematic analysis of 68 photovoice contributions. *South Afr J Oncol* 2017;**1**:8. doi:10.4102/sajo.v1i0.14

21 Rohleder P, Lyons AC. *Qualitative Research in Clinical and Health Psychology*. Macmillan International Higher Education 2014.

22 Freire P. Pedagogy of the Oppressed. 1968. *Trans Myra Bergman Ramos N Y Herder* 1972.

23 Smith JA. Evaluating the contribution of interpretative phenomenological analysis. *Health Psychol Rev* 2011;**5**:9–27. doi:10.1080/17437199.2010.510659

24 Keating F. Black men's conversations about mental health through photos. *Qual Soc Work* 2021;**20**:755–72. doi:10.1177/1473325020922293

25 Brunsden V, Goatcher J. Reconfiguring Photovoice for Psychological Research. *Ir J Psychol* 2007;**28**:43–52. doi:10.1080/03033910.2007.10446247

26 Charmaz K. Reconstructing grounded theory. *SAGE Handb Soc Res Methods* 2008;:461–78.

27 Freedman DA, Pitner RO, Powers MCF, *et al.* Using Photovoice to Develop a Grounded Theory of Socio-Environmental Attributes Influencing the Health of Community Environments. *Br J Soc Work* 2014;**44**:1301–21. doi:10.1093/bjsw/bcs173

For peer review only

28 Bradbury H, Reason P. Action Research: An Opportunity for Revitalizing Research Purpose and Practices. *Qual Soc Work* 2003;**2**:155–75. doi:10.1177/1473325003002002003

29 Using thematic analysis in psychology: Qualitative Research in Psychology: Vol 3, No 2. https://www.tandfonline.com/doi/abs/10.1191/1478088706QP063OA (accessed 25 Feb 2022).

30 Chew HSJ, Lopez V. Empowered to Self-Care: A Photovoice Study in Patients With Heart Failure. *J Transcult Nurs* 2018;**29**:410–9. doi:10.1177/1043659617745138

31 GLEESON K. Polytextual Thematic Analysis for visual data – pinning down the analytic. In: *Visual Methods in Psychology*. Routledge 2011.

32 Han CS, Oliffe JL. Photovoice in mental illness research: A review and recommendations. *Health (N Y)* 2016;**20**:110–26. doi:10.1177/1363459314567790

33 Tsang KK. Photovoice Data Analysis: Critical Approach, Phenomenological Approach, and Beyond. *Beijing Int Rev Educ* 2020;**2**:136–52. doi:10.1163/25902539-00201009

34 Experience-based investigation and codesign of approaches to prevent and reduce Mental Health Act use (CO-PACT): a protocol | BMJ Open. https://bmjopen.bmj.com/content/12/2/e060086.abstract (accessed 25 Feb 2022).

35 Sutton-Brown CA. Photovoice: A Methodological Guide. *Photogr Cult* 2014;**7**:169–85. doi:10.2752/175145214X13999922103165

36 Remembering One's Stay in Hospital: A Study in Photography, Recovery and Forgetting - Alan Radley, Diane Taylor, 2003. https://journals.sagepub.com/doi/abs/10.1177/1363459303007002872?casa_token=Ld7KzjvM3hYAAAAA:U7TJUWgeZddjF96WvPeiw9y0qJdX-h8a3DwmrLY1r-laaeY_dd7QOaOYbvqXSrvjnJ51mEf__K_2uQ (accessed 26 Feb 2022).

For peer review only

For peer review only

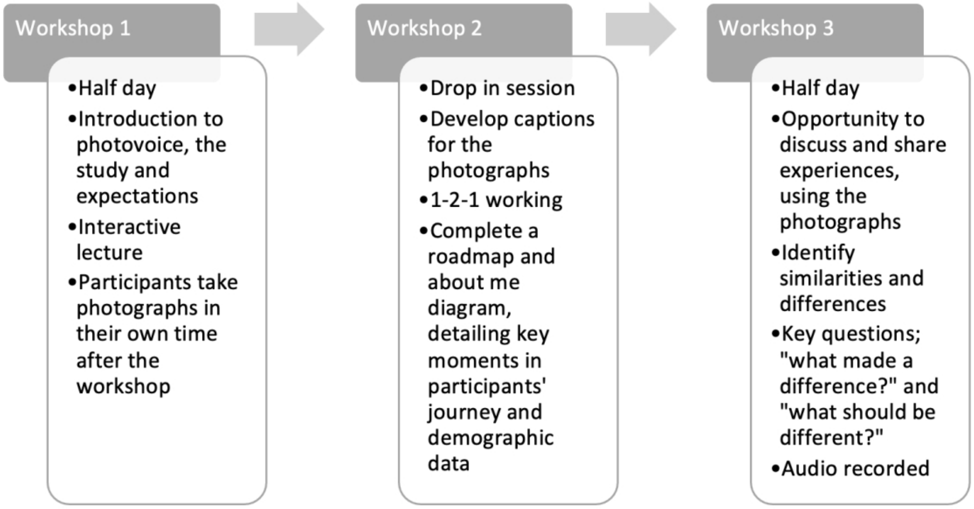

A brief description of content covered in a series of three photovoice workshops, undertaken with people who had experience of being detained under the Mental Health Act, conducted as part of the Co-PACT study.

274x145mm (144 x 144 DPI)
